# Altered grey matter volume in ‘super smellers’

**DOI:** 10.1007/s11682-018-0008-9

**Published:** 2018-12-07

**Authors:** Albert Wabnegger, Carina Schlintl, Carina Höfler, Andreas Gremsl, Anne Schienle

**Affiliations:** Clinical Psychology, BioTechMed Graz, University of Graz, Universitätsplatz 2, 8010 Graz, Austria

**Keywords:** Super smellers, Voxel-based morphometry, Insula, Hippocampus

## Abstract

‘Super smellers‘ are those individuals who show a heightened sense of smell. Data on the structural neuroanatomy of this phenomenon are still missing. A voxel-based morphometry study was conducted in order to compare gray matter volume (GMV) in specific brain regions of the olfactory network (piriform/entorhinal cortex, orbitofrontal cortex, insula, hippocampus) between 25 male ‘super smellers’ and 20 normosmic men. Participants were assigned to these groups based on their scores on a standardized olfactory performance test. Relative to normosmic men, ‘super smellers’ showed increased GMV in the anterior insula and in the hippocampus (dentate gyrus). These regions are crucial for the integration of olfactory information as well as odor learning and odor memory. Moreover, positive correlations between hippocampal volume and olfactory performance were detected in both groups. Future research should elaborate on how much of the observed neuroanatomical pattern of ‘super smellers’ is genetic and how much of it reflects experienced-based GMV increase.

**Electronic supplementary material:**

The online version of this article (10.1007/s11682-018-0008-9) contains supplementary material, which is available to authorized users.

## Introduction

Quantitative alterations in olfactory function can include both impairment and enhancement of performance. For example, the ability to detect odors can be reduced (hyposmia), it can be lacking for particular odorants despite the preserved ability to smell most other odors (specific anosmia), or it can be absent, which is the case in anosmia (Hummel et al. 2017). The causes of these changes are manifold, including sinonasal disease (e.g., sinusitis), infections (e.g., of the respiratory tract), exposure to toxins/drugs, tumors, neurological/neurodegenerative disease (e.g., Parkinson’s disease, dementia) and genetic conditions (congenital); however, there can also be idiopathic causes (without any connection to a medical diagnosis). In addition, olfactory dysfunction is associated with normal aging (for summaries see: Doty and Bromley 2007; Hummel et al. 2017).

In contrast, some individuals show an increased olfactory acuity (hyperosmia), as evidenced by a lowered odor threshold (Walker 1990). Hummel et al. (2017) described hyperosmia as a rare condition characterized by the ‘quantitatively increased ability to smell odors to an abnormal level’ (p. 3). Various somatic factors (e.g., migraine attacks) are associated with a heightened sense of smell (Blau and Solomon 1985). However, there is generally no known medical cause of hyperosmia (idiopathic origin; Doty and Bromley 2007).

Despite the fact that there are no clear criteria for diagnosing hyperosmia, it is generally agreed that heightened olfactory performance needs to be determined by standardized quantitative testing (Hummel et al. 2017). Most psychophysical tests of olfactory function assess threshold detection, identification, discrimination, and sometimes odor memory (for reviews see Doty 2015; Hummel et al. 2017). Odor threshold refers to the lowest concentration of an odorant that a person is able to perceive. A reliable assessment can be achieved by using a single staircase odor detection threshold test (e.g., Hummel et al. 2007). Here, the concentration of an odorant is (repeatedly) increased following trials on which a subject has failed to detect the odorant, and decreased following trials with a correct response. Relative to odor identification and classification, threshold is the most sensitive indicator of change (e.g., decline) in olfactory performance (Hummel et al. 2007).

Several voxel-based morphometry (VBM) studies have investigated the neuroanatomy of the central olfactory network in the context of normal as well as impaired olfactory function (e.g., Bitter et al. 2010a, b; Frasnelli et al. 2013; Han et al. 2017; Peng et al. 2013; Rombaux et al. 2006a, b; Seubert et al. 2013a, b; Yao et al. 2014, 2017). These studies have investigated whether olfactory performance, as indexed by the ability to identify, to classify (label), or to detect an odor (threshold), is associated with gray matter volume (GMV) in specific areas of the olfactory network. These areas include the olfactory bulb, the primary olfactory cortex (piriform cortex), the secondary olfactory cortex (orbitofrontal cortex (OFC), insula) and tertiary olfactory regions (e.g., hippocampus). It has been consistently shown that acquired loss of olfactory function (partial or complete) is associated with decreased GMV in the olfactory bulb, the piriform cortex (PC), the anterior cingulate cortex, the OFC, the insula and the cerebellum (Bitter et al. 2010a, b; Peng et al. 2013; Yao et al. 2014). However, in contrast to these results, Frasnelli et al. (2013) found patients with congenital anosmia to exhibit larger GMV in the entorhinal and piriform cortex. In healthy normosmic subjects, overall olfactory performance (as indexed by the sum score of the ‘Sniffin’ Sticks Test’ by Hummel et al. 2007) has been positively correlated with the volume of the OFC (Seubert et al. 2013a).

The current VBM study focused on the structural neuroanatomy of superior olfactory ability, since, to the best of our knowledge, no data are presently available on this topic. It was investigated whether healthy men with an outstanding olfactory performance (‘super smellers’) are characterized by increased brain volume in specific regions of the central olfactory system (piriform/entorhinal cortex, insula, hippocampus, OFC) relative to normosmic men. The analysis was restricted to male individuals since sex differences in GMV in the olfactory system as well as in olfactory performance have been observed (e.g., Garcia-Falgueras et al. 2006).

## Experimental procedures

### Sample

VBM data from 45 male University students (mean age: 25.4 years, SD = 5.9) were analyzed. Based on their olfactory performance (total score obtained in the Sniffin’ Sticks Test, Hummel et al. 2007) the men had been assigned to either a ‘super smeller’ group (*n* = 25) or a ‘normal smeller’ group (*n* = 20; see Table 1). Both groups did not differ in mean age and years of education (ps > .40). Any reported diagnoses of neurological, mental, or otorhinolaryngological disease known to be associated with olfactory dysfunctions led to exclusion from the sample. Furthermore, participants were medication naïve. All participants gave written informed consent. The study had been approved by the local ethical committee and was performed according to the guidelines of the Declaration of Helsinki 1975.

**Table 1:** Results of t-tests for the comparison of age, years of education and olfactory performance between super smellers and normal smellers (means, standard deviations, range, effect size)

	Super smellers		Normal smellers		t-test	Effect size
	*M* (*SD*)	*Range*	*M* (*SD*)	*Range*	*p*	*d*
Age	25.44 (6.63)	19–48	25.35 (5.11)	20–41	.960	0.01
Years of education	12.52 (1.29)	11–15	12.85 (1.46)	11–15	.427	0.24
Threshold	13.8 (2.8)	11–16	7.2 (1.4)	4.25–9.75	<.001	2.91
Discrimination	14.9 (1.2)	12–16	13.4 (1.3)	11–15	<.001	1.22
Identification	13.7 (1.5)	11–16	12.9 (1.5)	9–15	.037	0.55
Sum score (TDI)	42.4 (1.7)	41–46	33.4 (1.9)	30–37	< .001	4.86

### Procedure

A total of 161 male University students (M = 26.65 years; SD = 7.77) were recruited by advertisements at the University campus. We asked men who considered themselves as ‘super smellers’ to participate in the study. They were screened with the extended Sniffin’ Sticks Test (Burghart ltd. Instruments, Wedel, Germany), which is a clinically approved test of olfactory function including threshold, discrimination and identification assessment (Hummel et al. 2007). The odorants were presented to the blind-folded participants with pen-like odor dispensing devices. The olfactory detection threshold was assessed with 푛-butanol, which was presented in 16 dilutions in a staircase, three-alternative, forced-choice procedure. Odor discrimination ability was obtained by presenting 16 triplets of odorant pens (two pens contain the same odorant; the third pen contains a different odorant). The participants’ task was to detect the different odor. Odor identification was assessed by means of 16 common odors (e.g., coffee, cinnamon). Subjects identified the odors by selecting the best label from a list of four descriptors. Possible scores for the detection threshold range between 1 and 16 (with higher scores indexing lower thresholds), and for the other two subtests between 0 and 16. The scores for all three subtests were summed to obtain the Threshold-Detection-Identification (TDI) score.

Two groups labeled ‘super smellers’ (*n* = 25) and ‘normal smellers’ (*n* = 20) were created based on normative data reported by Hummel et al. (2007). A TDI score > 41 was considered the inclusion criterion for the ‘super smeller’ group (13 participants of this group had the highest possible threshold score of 16). TDI scores above 41 equal the 90th percentile for male individuals in the age range between 16 and 35 years. TDI scores followed a normal distribution in both groups (Shapiro-Wilk test; all *p* > .17). Men of the ‘normal smellers’ group had TDI scores between 30 and 37 (they were drawn from the screening sample to match in age and educational status with the ‘super smeller’ group). The comparison of both groups by means of one-tailed t-tests showed that super smellers scored higher on all three olfactory subtests (threshold, discrimination, identification) (see Table 1).

All participants completed the Brief Symptom Inventory (BSI; Franke 2000), which is a 53-item self-report instrument for psychological problems and their intensity during the last week (Cronbach’s alpha in the current sample = .93). The items are rated on 5-point scales (0 = ‘not at all’ to 4 = ‘extremely’). The BSI is composed of nine symptom dimensions: Somatization (e.g., chest pain, upset stomach); Obsessive-compulsive symptoms (e.g., repeated checking); Interpersonal Sensitivity (e.g., feelings of inferiority); Depression (e.g., feeling hopeless); Anxiety (e.g., nervousness); Hostility (e.g., having urges to harm someone); Phobic Anxiety (e.g., avoidance of certain places, objects or activities because of anxiety), Paranoid Ideation (e.g., the feeling that you cannot trust others) and Psychoticism (e.g., the idea that someone has power over your thoughts). In addition, a sum score (global index of distress; GSI) is computed. We selected the BSI for this study because previous research has shown that persons with impaired olfactory performance (e.g., anosmic/hyposmic patients; 10th percentile of TDI scores) tend to report more psychological problems than normosmic individuals (e.g., Kohli et al. 2016; Schienle et al. 2018). Now, we were interested whether superior olfactory performance (90th percentile of TDI scores) might be associated with the presence of specific symptoms of mental disorders as well. Finally, all men were asked to report previous and current diagnoses of neurological and mental disorders and diseases of the ear/nose, pharynx/larynx, head/neck, as well as intake of medication.

### Image acquisition and VBM analysis

T1-weighted scans were acquired using a 3-T Siemens Skyra with a 32-channel head coil (Siemens, Erlangen, Germany). The scanning parameters were as follows: voxel size: 0.88 × 0.88 × 0.88 mm; 192 transverse slices, FoV = 224 mm, slice thickness: 0.88 mm, TE = 1.89 ms, TR = 1680 ms; TI = 1000 ms, flip-angle = 8°. Structural scans were analyzed with the Computational Anatomy Toolbox (CAT12; r1113) implemented in SPM12 (v6906; Wellcome Trust Centre for Neuroimaging; http://www.fil.ion.ucl.ac.uk/spm/software/spm12/) for voxel-wise comparisons of GMV.

Prior to the normalization procedure, origin of each individual T1-weighted image was manually set to the anterior commissure. All analyses were carried out with default settings of the CAT12 toolbox. Afterwards structural images were segmented into gray and white matter. Additionally images were modulated to compensate for the effect of spatial normalization. Finally, voxels were resliced to: 1.5 × 1.5 × 1.5 mm. All segmented and modulated gray matter images showed a large homogeneity (*r* > 0.87), therefore none of the subjects had to be excluded from the sample. Manual quality control of segmented gray matter images revealed a good quality (M = 7.34, SD = .55; 1 = very poor quality, 9 = very good quality). Finally, gray matter images were smoothed with a Gaussian kernel with a full width at half maximum (FWHM) of 6 mm.

Statistical analyses were carried out using random effects models. Possible group differences were investigated by using a two-sample t-test with the covariates age and total intracranial volume as regressors of no interest. Furthermore, olfactory performance (TDI score) was correlated with GMV within the multiple regression approach implemented in SPM12. All covariates have been mean centered. All analyses were conducted with an absolute threshold of 0.2 to ensure that all analyses were restricted to gray matter.

We conducted voxel-intensity tests for the whole brain as well as for regions of interest (ROIs: piriform cortex, entorhinal cortex, insula, hippocampus, OFC; see Figure S1). For the ROI analyses, masks were used with a 25% threshold from the Harvard-Oxford cortical structural atlas (Center for Morphometric Analysis, MGH-East, Boston/MA, USA). Because masks for the piriform/ entorhinal cortex are not available in the Harvard-Oxford atlas, we used a piriform mask with a 50% threshold described by Seubert et al. (2013a, b). The entorhinal mask was taken from the anatomy toolbox (Eickhoff et al. 2005). The masks were resliced to a voxel size of 1.5 × 1.5 × 1.5 mm with the nearest neighbor function. The volumes (in mm^3^) for the ROIs were as follows: piriform cortex (L: 2153 mm^3^, R: 1701 mm^3^); insula (L: 9153 mm^3^, R: 9565 mm^3^); hippocampus (L: 6521 mm^3^, R: 5788 mm^3^); OFC (L: 13551 mm^3^, R: 11117 mm^3^), entorhinal cortex (L: 2001 mm^3^, R: 2420 mm^3^). The used ROI masks are displayed in the supplementary Figure 1. Results of the voxel intensity-based tests were considered statistically significant if *p* < 0.05 (corrected for family-wise error) and the voxel was part of a cluster with at least five contiguous voxels. All ROI results were small volume corrected. We did not correct each individual ROI for multiple testing to minimize Type II errors.

## Results

### Brief symptom inventory (BSI)

Both groups did not differ with regard to the scores on the BSI subscales and the total score (GSI; Table 2). The men reported comparable BSI scores as the construction sample reflecting no increased psychological distress (all ps > .16).

**Table 2:** Results of t-tests for the comparison of psychological distress between super smellers and normal smellers (means, standard deviations, effect sizes)

	Super smellers	Normal smellers	t-test	Effect size
	*M* (*SD*)	*M* (*SD*)	*p*	*d*
Somatization	.20 (.35)	.14 (.31)	.569	.17
Obsessive-compulsive	.39 (.30)	.62 (.49)	.085	.57
Interpersonal sensitivity	.35 (.44)	.44 (.77)	.633	.14
Depression	.23 (.33)	.43 (.69)	.246	.38
Anxiety	.25 (.25)	.26 (.36)	.957	.02
Hostility	.33 (.39)	.35 (.55)	.876	.05
Phobic anxiety	.08 (.13)	.14 (.36)	.481	.24
Paranoid ideation	.23 (.36)	.24 (.52)	.952	.02
Psychoticism	.14 (.25)	.25 (.45)	.324	.30
Global severity index	.25 (.20)	.32 (.37)	.444	.23

### VBM

#### Group comparison

**Fig. 1:**
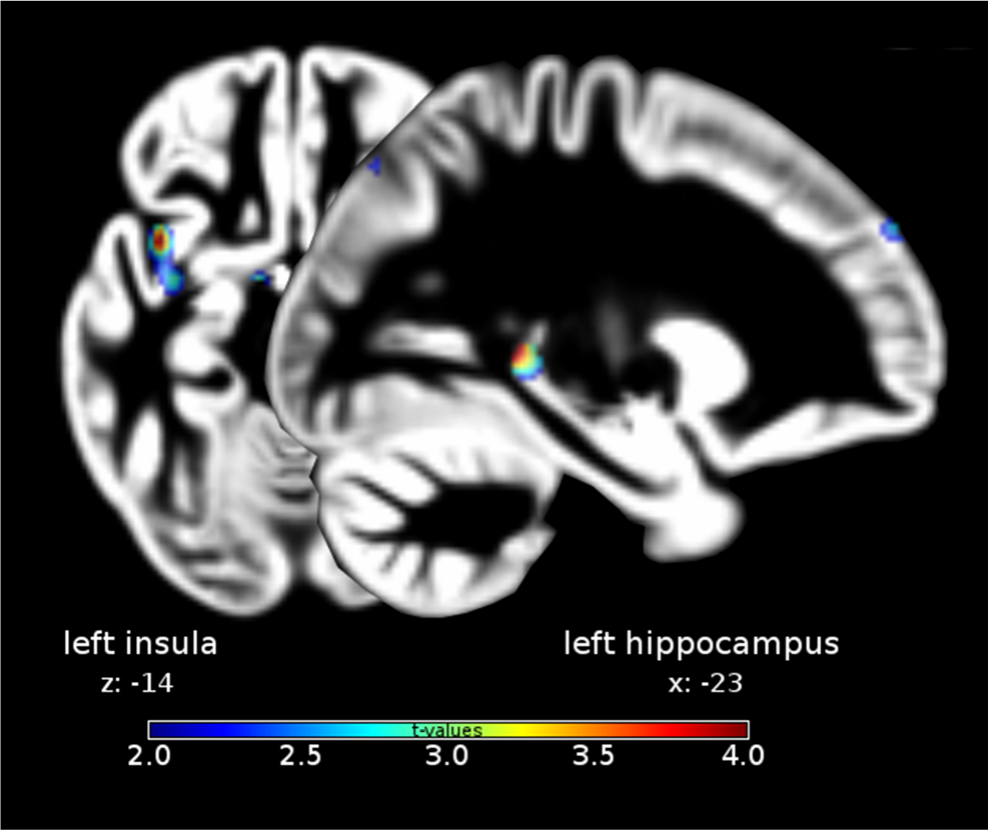
Increased grey matter volume in super smellers relative to normal smellers

‘Super smellers’ showed enhanced gray matter volume (GMV) in the left insula (MNI coordinates: x,y,z: −39,9,-14; *t* = 3.91; p(FWE) = 0.039; Cohen’s d = 1.22) and in the left hippocampus (x,y,z: −23,-41,3; *t* = 4.00; p(FWE) = 0.023, Cohen’s d = 1.25) relative to normosmic men (Fig. 1). Peaks of increased volume were found in the anterior part of the insula, and in the dentate gyrus.

When the TDI score was used as an additional covariate of no interest, group differences disappeared. This underlines that the localized brain volume increase in ‘super smellers’ was indeed based on olfactory function.

#### Correlations

**Fig. 2:**
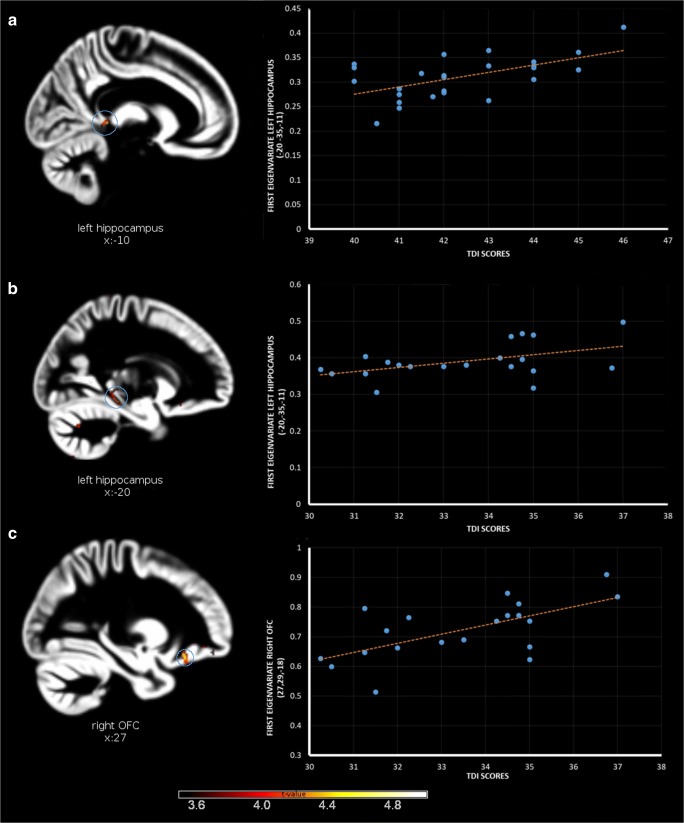
Correlations between olfactory performance and grey matter volume in regions of interest for super smellers (**a**) and normal smellers (**b**, **c**)

In the ‘super smeller’ group, the TDI scores were positively correlated with left hippocampal volume (x,y,z: −9,-38,2; *t* = 4.42; p(FWE) = 0.025; Cohen’s d = 1.93) (Fig. 2). In normosmic men, the TDI scores were positively correlated with GMV in the left hippocampus (x,y,z: −20,-35,-11; *t* = 4.24; p(FWE) = 0.042; Cohen’s d = 2.12) and in the right OFC (x,y,z: 27,28,-18, *t* = 4.52; p(FWE) = 0.047; Cohen’s d = 2.26).

Additionally, correlations between TDI scores and ROI activation were computed across all subjects. The results revealed that the TDI was positively correlated with GMV in the left and right hippocampus (left: x,y,z: −23,-41,3; *t* = 4.23; p(FWE) = .012; right: x,y,z: 26,-35,-2; *t* = 4.13; p(FWE) = .015) and in the left insula (x,y,z: −39,9,-14; *t* = 3.84; p(FWE) = .046). The associated correlation plots can be found in Figure S2.

## Discussion

The current voxel-based morphometry study compared gray matter volume (GMV) in brain regions associated with olfactory processing between ‘super smellers’ and normosmic men. To the best of our knowledge, this is the first investigation that has focused on the underlying neuronal correlates of healthy individuals with an above-average sensitivity to smell.

The ‘super smellers’ group was characterized by increased GMV in the insula and hippocampus. The insula is central for olfactory-gustatory processing and is considered part of the secondary olfactory cortex (Mazzola et al. 2017). It is an area of cross-modal integration (olfaction, gustation, trigeminal function; Lundström et al. 2011). Apart from its role in the representation of the chemical senses, the insula is also involved in general interoceptive awareness connecting homeostatic information from the body with higher-level cognitive processes (Craig 2009). This integration also involves affective processing (Paulus and Stein 2006). In particular, the anterior part of the insula has been shown to be involved in evaluation of the emotional quality of olfactory and trigeminal stimuli (particularly regarding negative valence and aversions in olfaction and trigeminal perception; Albrecht et al. 2009; Royet et al. 2003).

Functional neuroimaging studies have consistently demonstrated that the insular cortex contributes to different aspects of odor processing, such as odor quality coding (Veldhuizen et al. 2010) and odor detection (see meta-analysis by Seubert et al. 2013b). Moreover, a meta-analysis of gustatory and olfactory neuroimaging studies has shown that responses to odors occur in the same insula regions where taste responses are observed (Verhagen and Engelen 2006). It is important to note that a sense is seldom experienced in isolation; olfactory-gustatory perception usually works together to form a ‘flavor experience’.

The second brain structure with increased volume in ‘super smellers’ was the hippocampus, which receives input from the entorhinal cortex. The hippocampus is critical for odor-guided learning and memory (Rolls 2010). The volume increase observed in the ‘super smellers’ group was restricted to the dentate gyrus of the hippocampal formation, one of the few areas where adult neurogenesis can be observed in the mammalian brain (e.g., Amaral et al. 2008; Piatti et al. 2013). This implies enhanced neuroplasticity in this area, and may point toward the role which olfactory experience plays in heightened olfactory performance. Furthermore, in the current study, olfactory performance (as indexed by the TDI score) positively correlated with hippocampal volume. This was the case for the ‘super smellers’ group as well as for normosmic men. Somewhat similarly, in a previous investigation, Smitka et al. (2012) reported a small but significant correlation between hippocampal volume and odor threshold (*r* = 0.21) in normosmic volunteers. The authors of that study used manual contouring for volume quantification.

We were able to replicate the finding of a positive correlation between GMV in the OFC and general olfactory performance (as reflected by the TDI score) in the normosmic group (Seubert et al. 2013a). This brain region receives major projections from the piriform cortex and is involved in odor learning, cross-modal integration, and the affective evaluation of odors (Seubert et al. 2013a, b).

Additional correlation analyses across all subjects revealed positive relationships of GMV in the hippocampus and in the insula with TDI scores. This finding suggests an underlying linear trend in the total sample. However, computation of correlations in all subjects is not ideal because of the heterogeneity of the two selected groups of ‘super smellers’ and normal smellers. We also need to mention further limitations of the current study. In order to restrict sex-related variance, we only studied men. Therefore, the results cannot be generalized to women. In addition, the sample size of both groups was relatively small. However, the obtained TDI scores in the ‘super smellers’ group were outstanding; 13 out of 25 even achieved a perfect olfactory threshold score. Finally, it has to be noted that the current study did not apply correction for false positives (e.g., Bonferroni correction). It was attempted to minimize Type II errors, by the cost of increasing Type I errors. We believe that the large effect sizes (Cohen’s d: 1.22–2.26) underline that the present results provide a valuable approach for forthcoming studies investigating hyperosmia.

Such future studies now need to investigate the underlying mechanism of hyperosmia. How much of the observed neuroanatomical pattern in ‘super smellers’ is genetic and how much of it reflects increased exposure to odorants and effects of olfactory learning? As mentioned above, the hippocampus as part of the olfactory system shows considerable neuroplasticity. This plasticity might be linked to olfactory experience. In line with this hypothesis is a recent VBM study (Gellrich et al. 2017) that investigated brain-structural changes in hyposmic patients before and after 12 weeks of olfactory training. Following the intervention, the participants showed increases in gray matter volume in the hippocampus, thalamus, and cerebellum. It seems to be possible that olfactory training in normosmic individuals might produce similar hippocampal changes, and this should be investigated in a future study.

In conclusion, we found that high performance in a standardized olfactory test (upper 10%) was associated with increased GMV in secondary and tertiary regions of the central olfactory network (insula and hippocampus).

## Electronic supplementary material

Supplementary Figure S1 Region of interest masks (PNG 185 kb)

Supplementary Figure S2 Correlations between olfactory performance and grey matter volume in regions of interest across all subjects. Foot note: TDI = threshold-discrimination-identification score of the sniffin’ sticks test (PNG 666 kb)

High Resolution Image (TIF 103 kb)

## References

[CR1] Albrecht J, Kopietz R, Linn J, Sakar V, Anzinger A, Schreder T (2009). Activation of olfactory and trigeminal cortical areas following stimulation of the nasal mucosa with low concentrations of S(−)-nicotine vapor-an fMRI study on chemosensory perception. Human Brain Mapping.

[CR2] Amaral DG, Scharfman HE, Lavenex P (2008). The dentate gyrus, fundamental neuroanatomical organization (dentate gyrus for dummies). Progress in Brain Research.

[CR3] Bitter, T., Brüderle, J., Gudziol, H., Burmeister, H. P., Gaser, C., & Guntinas-Lichius, O. (2010a, 1347). Gray and white matter reduction in hyposmic subjects - a voxel-based morphometry study. *Brain Research*, 42–47. 10.1016/j.brainres.2010.06.003 20553879

[CR4] Bitter T, Gudziol H, Burmeister HP, Mentzel HJJ, Guntinas-Lichius O, Gaser C (2010). Anosmia leads to a loss of gray matter in cortical brain areas. Chemical Senses.

[CR5] Blau JN, Solomon F (1985). Smell and other sensory disturbances in migraine. Journal of Neurology.

[CR6] Craig AD (2009). How do you feel—Now? The anterior insula and human awareness. Nature Reviews. Neuroscience.

[CR7] Doty RL (2015). Olfactory dysfunction and its measurement in the clinic. World Journal of Otorhinolaryngology-Head and Neck Surgery.

[CR8] Doty R. L., & Bromley S. M. (2007) Chapter 7. Cranial nerve I: Olfactory nerve. In C. G. Goetz (Ed.), *Textbook of Clinical Neurology*. Philadelphia: Saunders.

[CR9] Eickhoff SB, Stephan KE, Mohlberg H, Grefkes C, Fink GR, Amunts K, Zilles K (2005). A new SPM toolbox for combining probabilistic cytoarchitectonic maps and functional imaging data. Neuroimage.

[CR10] Franke GH (2000). Brief symptom inventory - deutsche version manual.

[CR11] Frasnelli J, Fark T, Lehmann J, Gerber J, Hummel T (2013). Brain structure is changed in congenital anosmia. Neuroimage.

[CR12] Garcia-Falgueras A, Junque C, Giménez M, Caldú X, Segovia S, Guillamon A (2006). Sex differences in the human olfactory system. Brain Research.

[CR13] Gellrich Janine, Han Pengfei, Manesse Cedric, Betz Amelie, Junghanns Anne, Raue Claudia, Schriever Valentin A., Hummel Thomas (2017). Brain volume changes in hyposmic patients before and after olfactory training. The Laryngoscope.

[CR14] Han P, Whitcroft KL, Fischer J, Gerber J, Cuevas M, Andrews P, Hummel T (2017). Olfactory brain gray matter volume reduction in patients with chronic rhinosinusitis. International Forum of Allergy Rhinology.

[CR15] Hummel T, Kobal G, Gudziol H, Mackay-Sim A (2007). Normative data for the "Sniffin' sticks" including tests of odor identification. Odor discrimination. And olfactory thresholds, an upgrade based on a group of more than 3.000 subjects. European Archives of Oto-Rhino-Laryngology.

[CR16] Hummel T, Whitcroft KL, Andrews P, Altundag A, Cinghi C, Costanzo RM (2017). Position paper on olfactory dysfunction. Rhinology Supplement.

[CR17] Kohli P, Soler ZM, Nguyen SA, Muus JS, Schlosser RJ (2016). The association between olfaction and depression: A systematic review. Chemical Senses.

[CR18] Lundström JN, Boesveldt S, Albrecht J (2011). Central processing of the chemical senses, an overview. ACS Chemical Neuroscience.

[CR19] Mazzola L, Royet JP, Catenoix H, Montavont A, Isnard J, Mauguière F (2017). Gustatory and olfactory responses to stimulation of the human insula. Annals of Neurology.

[CR20] Paulus MP, Stein MB (2006). An insular view of anxiety. Biological Psychiatry.

[CR21] Peng P, Gu H, Xiao W, Si LF, Wang JF, Wang SK (2013). A voxel-based morphometry study of anosmic patients. British Journal of Radiology.

[CR22] Piatti VC, Ewell LA, Leutgeb JK (2013). Neurogenesis in the dentate gyrus, carrying the message or dictating the tone. Frontiers in Neuroscience.

[CR23] Rolls ET (2010). A computational theory of episodic memory formation in the hippocampus. Behavioural Brain Research.

[CR24] Rombaux P, Mouraux A, Bertrand B, Nicolas G, Duprez T, Hummel T (2006). Olfactory function and olfactory bulb volume in patients with postinfectious olfactory loss. Laryngoscope.

[CR25] Rombaux P, Mouraux A, Bertrand B, Nicolas G, Duprez T, Hummel T (2006). Retronasal and orthonasal olfactory function in relation to olfactory bulb volume in patients with posttraumatic loss of smell. Laryngoscope.

[CR26] Royet JP, Plailly J, Delon-Martin C, Kareken DA, Segebarth C (2003). fMRI of emotional responses to odors, influence of hedonic valence and judgment, handedness, and gender. Neuroimage.

[CR27] Schienle A, Wolf A, Tomazic PV, Ille R (2018). Affective personality traits in olfactory dysfunction: The role of dysthymia and arousal. Chemosensory Perception.

[CR28] Seubert J, Freiherr J, Frasnelli J, Hummel T, Lundström JN (2013). Orbitofrontal cortex and olfactory bulb volume predict distinct aspects of olfactory performance in healthy subjects. Cerebral Cortex.

[CR29] Seubert J, Freiherr J, Djordjevic J, Lundström JN (2013). Statistical localization of human olfactory cortex. Neuroimage.

[CR30] Smitka M, Puschmann S, Buschhueter D, Abolmaali N, Hummel T (2012). Is there a correlation between hippocampus and amygdala volume and olfactory function in healthy subjects?. NeuroImage.

[CR31] Veldhuizen, MG., Nachtigal, D., Teulings, L., Gitelman, D.R., Small, D.M. (2010). The insular taste cortex contributes to odor quality coding. *Frontiers in Human Neuroscience, 4*. 10.3389/fnhum.2010.00058 PMC2917218 20700500

[CR32] Verhagen JV, Engelen L (2006). The neurocognitive bases of human multimodal food perception, sensory integration. Neuroscience and Biobehavioral Reviews.

[CR33] Walker HK (1990). Clinical methods, the history. Physical. And laboratory examinations.

[CR34] Yao L, Pinto JM, Yi X, Li L, Peng P, Wei Y (2014). Gray matter volume reduction of olfactory cortices in patients with idiopathic olfactory loss. Chemical Senses.

[CR35] Yao Linyin, Yi Xiaoli, Pinto Jayant Marian, Yuan Xiandao, Guo Yichen, Liu Yifan, Wei Yongxiang (2017). Olfactory cortex and Olfactory bulb volume alterations in patients with post-infectious Olfactory loss. Brain Imaging and Behavior.

