# Supplementary figures and images for: Altered grey matter volume in ‘super smellers’
Source: Brain Imaging Behav. 2018 Dec 7;13(6):1726–32. doi: 10.1007/s11682-018-0008-9 (PMC6904410; doi:10.1007/s11682-018-0008-9)

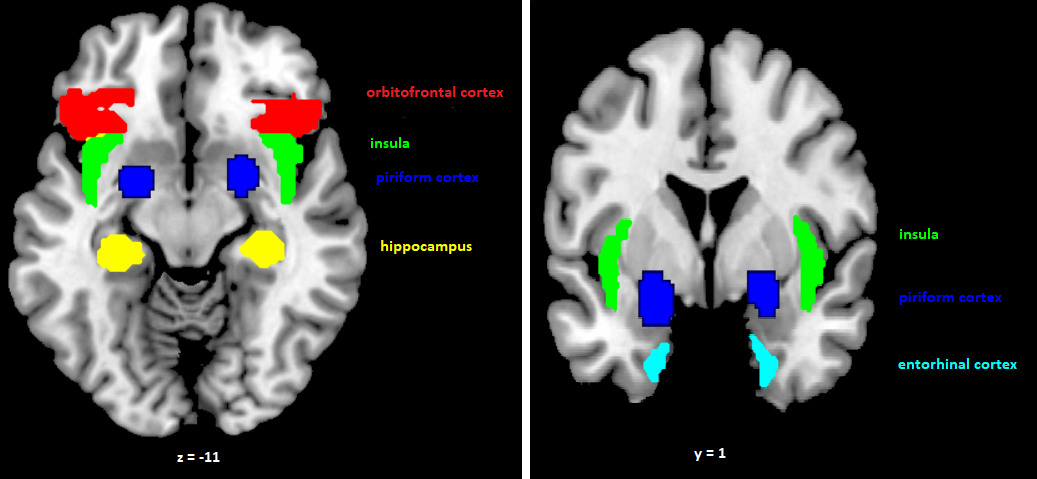

Supplement: Supplementary file 1 — Region of interest masks (PNG 185 kb) [file 11682_2018_8_MOESM1_ESM.png]

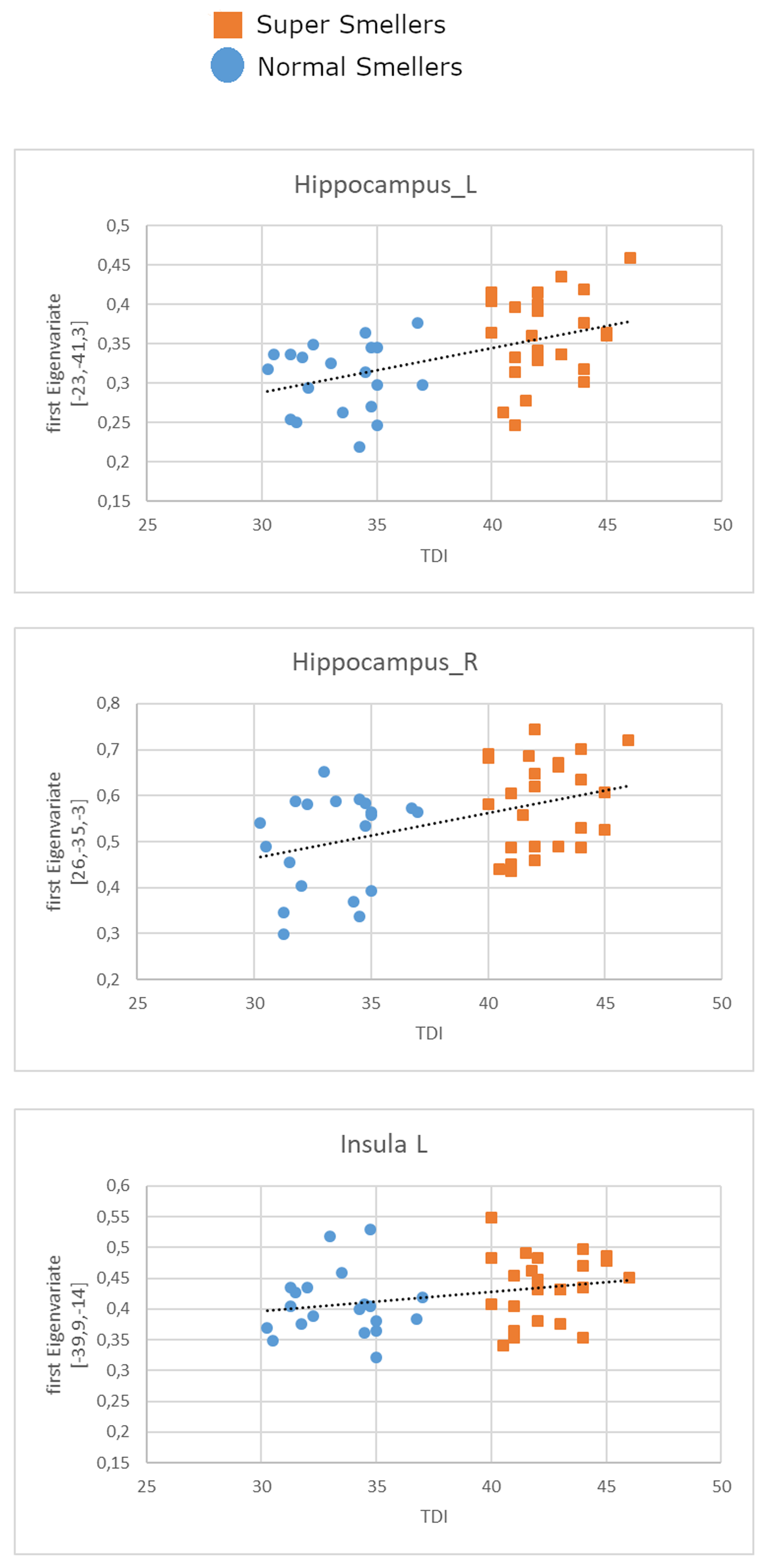

Supplement: Supplementary file 2 — Correlations between olfactory performance and grey matter volume in regions of interest across all subjects. Foot note: TDI = threshold-discrimination-identification score of the sniffin’ sticks test (PNG 666 kb) [file 11682_2018_8_Fig3_ESM.png]

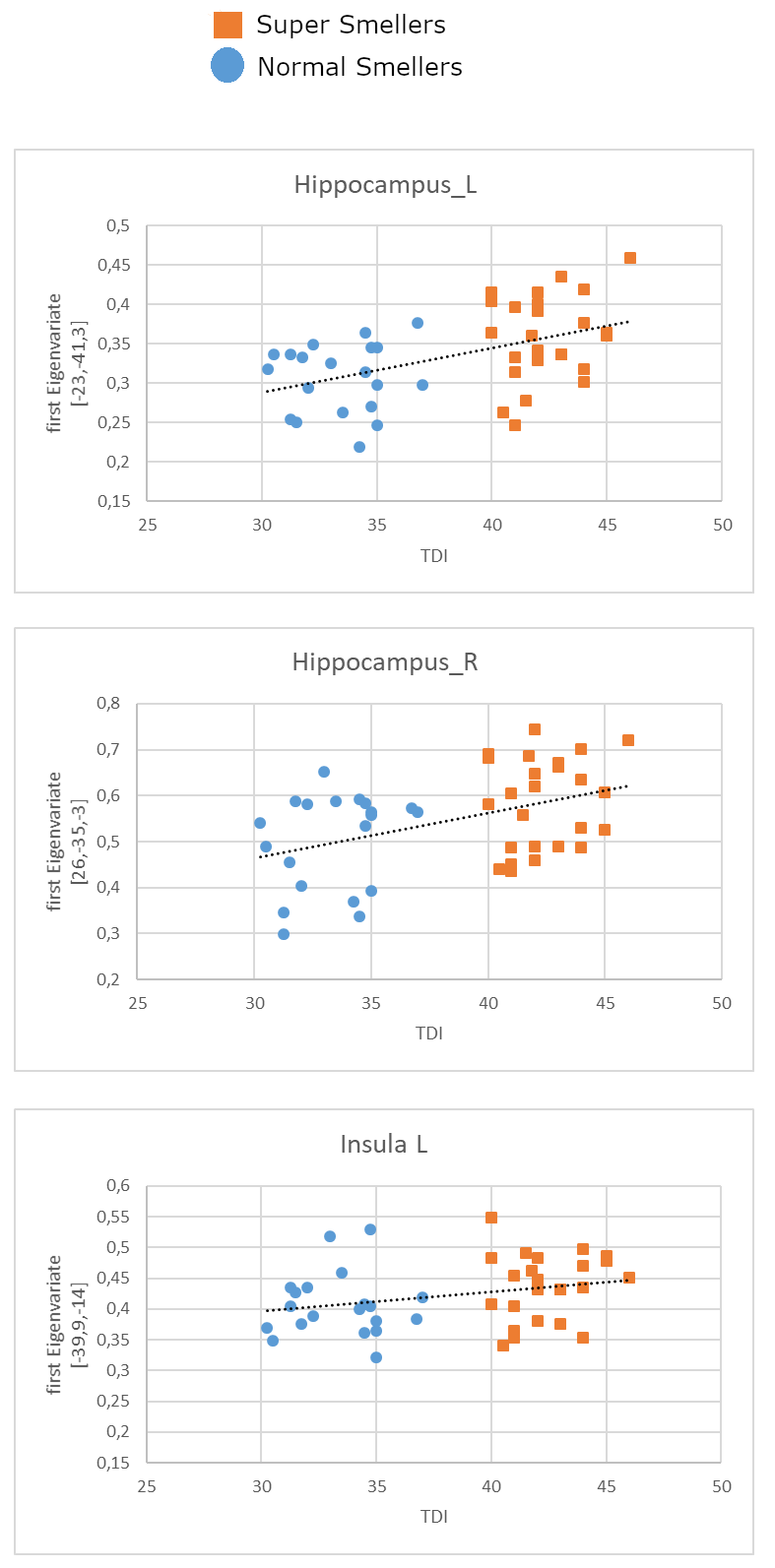

Supplement: Supplementary file 3 — High Resolution Image (TIF 103 kb) [file 11682_2018_8_MOESM2_ESM.tif]
